# LPS-Induced Systemic Neonatal Inflammation: Blockage of P2X7R by BBG Decreases Mortality on Rat Pups and Oxidative Stress in Hippocampus of Adult Rats

**DOI:** 10.3389/fnbeh.2019.00240

**Published:** 2019-11-06

**Authors:** Clivandir Severino da Silva, Michele Longoni Calió, Amanda Cristina Mosini, Jaime Moreira Pires, Débora da Silva Bandeira Rêgo, Luiz E. Mello, Ana Teresa Figueiredo Stochero Leslie

**Affiliations:** ^1^Departamento de Fisiologia, Universidade Federal de São Paulo—UNIFESP, São Paulo, Brazil; ^2^Departamento de Bioquímica, Universidade Federal de São Paulo—UNIFESP, São Paulo, Brazil; ^3^D’Or Institute for Research and Education (IDOR), Rio de Janeiro, Brazil; ^4^Departamento de Pediatria, Universidade Federal de São Paulo—UNIFESP, São Paulo, Brazil

**Keywords:** P2X7 receptor, brilliant blue G, lipopolysaccharide, neonate, inflammation, nervous system

## Abstract

Neonatal lipopolysaccharide (LPS) exposure-induced brain inflammation has been associated to neuronal injury and facilitates the development of models of neurological disorders in adult rats. The P2X7 receptor (P2X7R) plays a fundamental role in the onset and maintenance of the inflammatory cascade. Brilliant blue G (BBG), a P2X7R antagonist, has been shown to effectively promote neuroinflammatory protection. Here, we have investigated the long-term effects of the neonatal systemic inflammation on hippocampal oxidative stress, anxiety behavior and pain sensitivity in adulthood. We hypothesized that P2X7R blockade is able to modulate the effects of inflammation on these variables. Male and female rat pups received LPS and/or BBG solution intraperitoneally on the 1st, 3rd, 5th and 7th postnatal days. The survival rate and body weight were evaluated during the experimental procedures. The animals were submitted to behavioral tests for anxiety (elevated plus maze, EPM) and nociception (hot-plate and tail-flick) and the oxidative stress was measured by superoxide production in the dentate gyrus of the hippocampus using dihydroethidium (DHE) probe. BBG increased the survival rate in LPS-treated rats. No significant differences were found regarding anxiety behavior and pain sensitivity between the experimental groups. Systemic neonatal inflammation leads to a higher production of superoxide anion in the dentate gyrus of the hippocampus in adulthood and BBG inhibited that effect. Our data suggest that blocking the activation of the P2X7R during neonatal systemic inflammation may have a potential neuroprotective effect in adulthood.

## Introduction

Perinatal inflammation is a major contributor to brain injury in preterm infants. The immune system response induced by inflammation, in especial during the neonatal period, where the developing brain is vulnerable to adverse events, may play an important role in the brain function, leading to long-term neurological and psychiatric disorders, such as schizophrenia, autism, bipolar disorder and major depression (Peng et al., 2019).

Strong evidence has shown that systemic inflammation can activate microglia, leading to neuronal death and a significant increased extracellular adenosine triphosphate (ATP) concentration (Raetz and Whitfield, 2002; Fan et al., 2005, 2008, 2011; Ling et al., 2006). Activated microglia secretes high levels of inflammatory mediators, including tumor necrosis factor-α (TNF-α), interleukin-1β (IL-1β) and reactive oxygen species (ROS; Qian et al., 2010). These inflammatory mediators impair neurons and further activate microglia, which promotes further inflammation and neurodegeneration (Gao et al., 2002; Politis et al., 2012). The free radical production associated with the microglial cells activation, mainly superoxide radical, can trigger brain damage (Lambert and Brand, 2009).

Superoxide can be synthesized by the action of nicotinamide adenine dinucleotide phosphate (NADPH) oxidase 2 (NOX2). This enzyme is part of the family proteins constitute by seven members specialized in the synthesis of ROS, being a multicomponent enzyme system composed of membrane-bound (p22-phox and gp91-phox) and cytoplasmic subunits (p40-phox, p47-phox, and p67-phox; Bedard and Krause, 2007).

Gilles first documented in 1976 the vulnerability of the newborn brain to inflammation using the lipopolysaccharide (LPS; Gilles et al., 1976) and since then, several models have been published. Neonatal exposure to LPS, an endotoxin component of the cell wall of gram-negative bacteria (Raetz and Whitfield, 2002), has been considered a model of inflammation in which there is increased risk of neural disorders (Ling et al., 2006; Fan et al., 2011) as well as of functional disability, such as sensory, motor, emotional and cognitive impairment in juvenile rats (Fan et al., 2005, 2008). Also, LPS treatment causes a dose-dependent increase in pro-inflammatory cytokines (Peng et al., 2019). Recent data has demonstrated that the neonatal exposure to LPS may lead to persistent hippocampal injury (Wang et al., 2013).

The chemical composition of LPS varies according to the bacterial serotype, but its general structure consists of a hydrophilic polysaccharide domain linked to a hydrophobic lipid component, called lipid A (Raetz and Whitfield, 2002; Fan et al., 2011). LPS is not recognized by the host’s immune system while anchored to the outer bacterial membrane. The proliferation and bacterial lysis, however, cause the release of LPS from the membrane and the exposure of lipid A, which is recognized by the immune system (Raetz and Whitfield, 2002; Fan et al., 2005, 2011; Ling et al., 2006).

P2X7 receptor (P2X7R), a purinergic ATP-binding receptor, has been identified as a key player in the neuroinflammatory cascade controlling the onset and progression of a wide range of neurological conditions (Parvathenani et al., 2003; Monif et al., 2009; Ulrich et al., 2012; Burnstock and Volonté, 2012; Sperlágh and Illes, 2014). The expression of P2X7R is enhanced in several types of brain diseases, in which the presence of activated microglia is a concurrent feature (Sperlágh and Illes, 2014). The outflow of ATP from damaged and dead cells leads to the proliferation and activation of microglia by P2X7R, which also stimulates the production of superoxide (Parvathenani et al., 2003; Monif et al., 2009).

Díaz-Hernández et al. (2009) demonstrated that administration of brilliant blue G (BBG), a P2X7R antagonist, in an animal model of Huntington’s disease in mice, prevented neuronal apoptosis and attenuated body weight loss and motor-coordination deficits. In another study, BBG injection after spinal cord injury resulted in the recovery of motor function (Peng et al., 2009). The blockade of P2X7R-mediated activity by BBG also showed to be neuroprotective in an animal model of Alzheimer’s disease (Ryu and McLarnon, 2008).

Here, we hypothesized that P2X7R blockade in the LPS model could reduce the hippocampal oxidative stress and also would attenuate anxiety and nociceptive responses in adulthood resulting in neonate inflammation.

## Materials and Methods

### Animals

Rats (*Wistar norvegicus)* were obtained and maintained in the Center for the Development of Experimental Models in Medicine and Biology (CEDEME) of Universidade Federal de São Paulo (UNIFESP). A total of 121 Wistar rat pups were used in the present study. Twenty-one days after birth the animals were separated by sex. All animals were housed in polypropylene cages under standard pathogen-free conditions (light/dark cycle 12 h/12 h, under constant room temperature at 22 ± 2°C, food, and tap water *ad libitum*). All of the experimental procedures were conducted according to international regulations of the National Institutes of Health, Guide for the Care and Use of Laboratory Animals (NIH Publication No. 8023), revised 2011, and approved by the internal Ethics Committee on Animal Research of UNIFESP (approval n° 4591030915).

### Drug Administration

On the 1st day after birth (post natal day, PND 1) the pups were randomly allocated into four groups as described below:

1. NAIVE = no drug administration;
2. SAL + SAL = two injections of sterile 0.9% saline solution 5 mL/Kg;
3. SAL + LPS = injection of sterile 0.9% saline solution 5 mL/Kg and injection of LPS, 1 mg/Kg dissolved in saline;
4. BBG + LPS = injection of BBG 50 mg/Kg (Feng et al., 2015) dissolved in water and injection of LPS 1 mg/Kg dissolved in saline.

Drugs: LPS (Sigma L-2630; *Escherichia coli*, 0111:B4); BBG (Sigma-Aldrich B0770). All of the drugs were administered intraperitoneally (i.p.) on PND1, 3, 5 and 7 with a 30 min interval between injections. The animals were continuously monitored during handling, administration of the drugs and after the procedures and all events and observations from the pups were recorded. Body weight was assessed on PND1, 10, 21, 45 and 89. The NAIVE and SAL + SAL groups were initially compared using the Fisher’s exact test or the *t*-Student test and the results of the two groups were pooled as no statistically significant differences existed between both. The resulting group is reported as CONTROL.

It is known that a single systemic injection of LPS is used to reproduce acute systemic inflammation, whereas multiple injections mimic a chronic inflammatory condition (Simons and Tibboel, 2006; Püntener et al., 2012; Rousset et al., 2013; Dinel et al., 2014; Ming et al., 2015). Although LPS penetration into the central nervous system (CNS) is low, a single systemic injection is sufficient to trigger acute neuroinflammation (Elmquist and Flier, 2004; Sachot et al., 2004; Spencer et al., 2006). However, repetitive systemic use of LPS supports the activated microglial phenotype and causes changes in *blood*–*brain barrier*, increasing the penetration of LPS into the CNS and mobilizing other elements involved in the inflammatory response, and neuronal death (Schwartz et al., 2000; Benatti et al., 2009; Cervetto et al., 2013). For these reasons, repeated injections of LPS in alternate days was used in our model, in order to mimic a process of persistent neonatal inflammation. We created a new protocol aiming to reproduce a persistent inflammatory process in the first week of life and the dose of LPS was chosen based on the study by Okuyama et al. (2013).

### Behavioral and Nociceptive Tests

In all experiments, the animals were observed in a blind manner as to which group the animals belonged to, and the apparatus was cleaned with a 5% alcohol solution after each session. In order to verify the influence of estrous cycle on treatment, female rats were evaluated according to the stage of the estrous cycle (see Supplementary Material).

#### Elevated Plus-Maze

The elevated plus maze (EPM) test evaluates anxiety-like behavior and combines natural preferences of rodents for dark spaces and aversions to illuminated, open and or elevated areas (Lezak et al., 2017). The EPM test was performed on PND80. As described by Pellow et al. (1985), the EPM consists of an apparatus made of wood, with two open arms (50 × 10 × 1 cm), and two closed arms (50 × 10 × 50 cm) with an open roof and arranged such that the two arms are opposite and perpendicular to each other, elevated 50 cm above the floor. The animals were individually placed at the center of the maze, facing towards one of the open arms and observed for 5 min. The ratio of time spent in the open arms, the ratio of time spent in central platform, the ratio of entries into open arms, the total number of entries into the arms (enclosed plus open), and the traveled distance were calculated. The measures for EPM test were taken using a camera (Panasonic; model WV-CP504) and analyzed with the program EthoVision (Noldus, 7.0).

#### Hot-Plate

The hot plate test evaluates pain by the supra-spinal pathways (Woolfe and Macdonald, 1943; Eddy and Leimbach, 1953). Reaction latency to the hot-plate was measured at PND82. Rats were placed individually on a hot-plate metallic surface (Ugo Basile S.R.L, model 35100-001) maintained at 55° ± 0.2°C. The latency time was measured by the time between placement of the animal on the hot-plate and the occurrence of the first sign of nociception, paw licking, flinching or jump response to avoid the heat. Reaction time was recorded and the animal immediately removed from the hot plate. A cut-off period of 30 s was set to avoid tissue damage to the paws. The values were taken manually.

#### Tail-Flick

The tail-flick test evaluates spinal reflex that can be an indication of pain (D’Amour and Smith, 1941; Cartens and Wilson, 1993). The nociceptive response was also evaluated by recording the latency to withdrawal of the tail in response to heat on PND84. Rats were habituated to handling and to being inserted into plastic cylindrical tubes before the experimental procedures. The tails of the rats were immersed in heated water. The heat intensity was set by adjusting the temperature at 52° ± 1°C. When a withdrawal response occurred, the stimulus was terminated and the response latency was measured. A cut-off time of 30 s was used to avoid tissue damage. The values were taken manually.

### Tissue Preparation

The animals were maintained until the PND89, when euthanasia was performed. Animals were deeply anesthetized with a lethal dose of ketamine cocktail 80 mg/Kg ketamine (100 mg/mL—Syntec), 15 mg/Kg xylazine (20 mg/mL—Syntec) and 1 mg/Kg acepromazine (2 mg/mL—Vetnil) and intracardially perfused (infusion pump Cole-Parmer/Masterflex; model 7518-00), through the ascending aorta with 0.9% saline solution and 4% cold paraformaldehyde. The brain tissues were fixed in 4% paraformaldehyde for 24 h followed by immersion in a 30% sucrose in 0.1 M phosphate buffer at 4°C for 72 h. Brain tissues were frozen in O.C.T. compound (A.O. Company, Milwaukee, WI, USA), and the organs were cut into 30 μm coronal sections on a cryostat (HYRAX C25 cryostat, Zeiss). The sections were stored in a cryoprotectant solution (30% sucrose, 30% ethylene glycol, 0.1 M phosphate buffer) at −80°C until processing for superoxide detection. We decided to select the dentate gyrus of the hippocampus because this is the most sensitive region to damage and neuronal death caused by oxidative stress.

### Evaluation of ROS Production

#### Detection of Superoxide

Superoxide was detected with the oxidative fluorescent probe DHE (dihydroethidium; Molecular Probes, CA, USA), which was oxidized to 2-hydroxyethidium, which then produced red fluorescence. Double staining was performed to assess the presence of DHE in three types of brain cells that express neuronal nuclear protein (NeuN) or glial fibrillary acid protein (GFAP) or ionized calcium binding adaptor molecule 1 (Iba1) as described below.

Tissue samples were washed three times with 0.1 M phosphate buffer for 5 min. Brain sections were incubated in a light-protected humidified chamber with 5 μM DHE. Cell nuclei were stained with nuclear tracer DAPI (4′,6-diamidino-2-phenylindole; 5 μM; Sigma-Aldrich). Stained slides were examined and imaged using a confocal microscope (Leica SP8 Lightning, Leica Microsystems with LAS × Lite software). Fluorescence was detected with 510–560 nm excitation and 590 nm emission filters. The results are expressed as the DHE/DAPI ratio. ImageJ was used for quantification of the red emission signal and pixilation analysis. The amount of red emission signal was normalized with DAPI. Pixilations were analyzed in four acquired non-overlapping images (10 stacks) per slice of each animal, being that six hippocampal slices per animal were analyzed.

#### gp91-phox/NOX 2 Expression

Immunofluorescence was used to detect gp91-phox subunit expression. Also, double immunofluorescence staining was performed to assess expression of gp91-phox in three types of brain cells, that express NeuN, GFAP and Iba1. The sections were incubated with 3% H_2_O_2_ and then incubated 2% normal blocking bovine serum. Next, the sections were incubated with primary antibody anti-gp91-phox (Novus Biologicals NBP2-13037; 1:200), anti-NeuN (Millipore MAB377; 1:500), anti-GFAP (Sigma-Aldrich G3893; 1:500) and/or anti-Iba1 (Abcam ab5076; 1:500) in room temperature. One day later, the sections were rinsed and incubated with secondary conjugated antibody Alexa Fluor 488 (Invitrogen A11034; Life Technologies A21202e A11055; 1:500), Alexa Fluor 546 (Invitrogen A11056; 1:500) and/or Alexa Fluor 594 (Invitrogen A21203; 1:500) and DAPI (5 μM; Sigma-Aldrich) for nuclear staining for 2 h. Stained slides were examined and imaged using a confocal microscope (Zeiss Axiovert 100 M; Carl Zeiss, Germany; connected to an LSM 810 Confocal Laser Scanning System or Leica SP8 Lightning, Leica Microsystems with LAS × Lite software). The results are expressed as the NOX2/DAPI ratio. ImageJ was used for quantification and pixilation analysis. The amount of green emission signal was normalized with DAPI. Pixilations were analyzed in two acquired non-overlapping images (five stacks) per slice of each animal, being that one hippocampal slice per animal was analyzed.

### Statistical and Data Analysis

Pearson’s Chi-squared and Fisher’s exact tests were applied to analyze the mortality rates in the groups or to compare males/females. The results were expressed as a percentage. When pertinent, one or two-way analysis of variance (ANOVA) followed by the Tukey’s correction *post hoc* test was used for multiple comparisons of groups vs. sex or groups (females only) vs. estrous cycle. The data were presented as mean ± SEM (standard error of the mean). A *p*-value < 0.05 was considered significant. Statistical analysis was performed using SPSS software (20.0.0), *Statistica* 13 software and GraphPad Prism 5.0.

## Results

### Effects of Neonatal LPS and BBG-Treatment on Mortality

**Figure 1:**
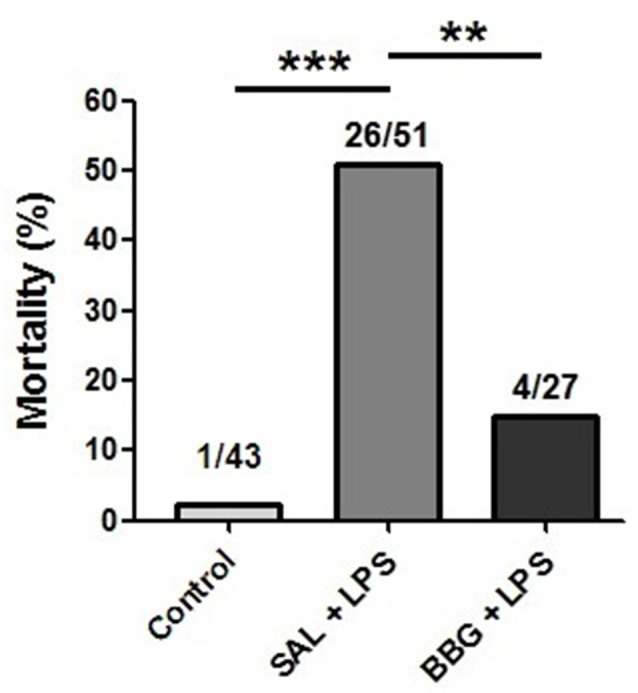
Effects of neonatal lipopolysaccharide (LPS) and brilliant blue G (BBG)-treatment on the mortality. BBG increased survival rate in LPS-treated rats. Data on bars represent percentage of dead animals (numbers listed above bars). **p < 0.01, ***p < 0.001.

Survival rates were 97.7% in the control group, 49% in SAL+LPS-treated group and 85.2% in BBG+LPS-treated group, with rats dying between PND1 and 11. The significantly higher mortality rate after LPS (*p* < 0.001; control group vs. SAL+LPS group) was not seen when the BBG treatment was associated (*p* = 0.002; SAL + LPS group vs. BBG+LPS group; Figure 1). No significant difference was observed in the mortality rate between males and females (*p* = 0.323). The LPS-treated pups demonstrated a “sick” appearance when compared to the other experimental group, showing pallor due to peripheral vasoconstriction, poor growth, lethargy and less mobility (see Supplementary Figure S1).

### Effects of Neonatal LPS and BBG-Treatment on the Body Weight Gain

**Figure 2:**
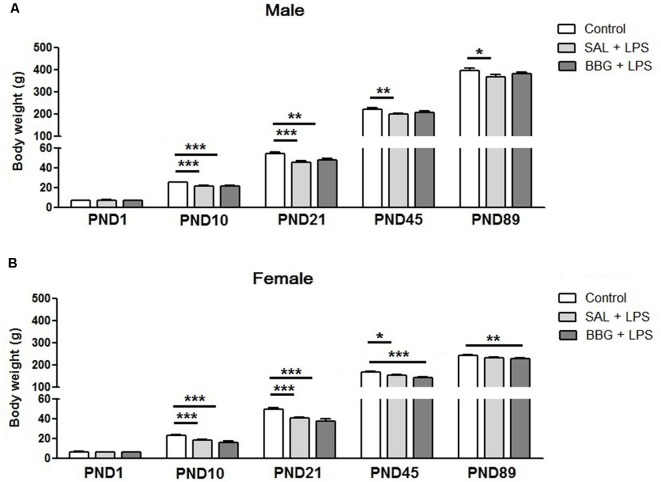
Effects of neonatal LPS and BBG-treatment on the body weight gain. The development of body weight was measured on PND1, 10, 21, 45 and 89 for male **(A)** and female **(B)** animals. LPS induced a decrease in weight gain. On the other hand, BBG induces increase in weight gain in male and a decrease in female. Data are expressed as mean ± standard error of the mean (SEM). *p < 0.05, **p < 0.01, ***p < 0.001.

There was no difference between the groups with respect to body weight on PND1 (males, *p* = 0.346; females, *p* = 0.414). However, significant differences were observed for males on PND10 (*p* < 0.001), SAL+LPS and BBG+LPS groups presented lower body weight when compared to control group; on PND21 (*p* < 0.001), SAL+LPS and BBG+LPS groups presented lower body weight when compared to control group; on PND45 (*p* = 0.008), SAL+LPS group presented lower body weight when compared to control group and on PND89 (*p* = 0.048), SAL + LPS group when compared to control group (Figure 2A).

We also observed significant differences for females on PND10 (*p* < 0.001), SAL+LPS and BBG+LPS groups presented lower body weight when compared to control group; on PND21 (*p* < 0.001), SAL+LPS and BBG+LPS groups presented lower body weight when compared to control group; on PND45 (*p* < 0.001), SAL+LPS and BBG+LPS groups presented lower body weight when compared to control group and on PND89 (*p* = 0.006), BBG+LPS group presented lower body weight when compared to control group (Figure 2B).

### Effects of Neonatal LPS and BBG-Treatment on the Behavioral and Nociceptive

#### Elevated Plus-Maze Test

**Figure 3:**
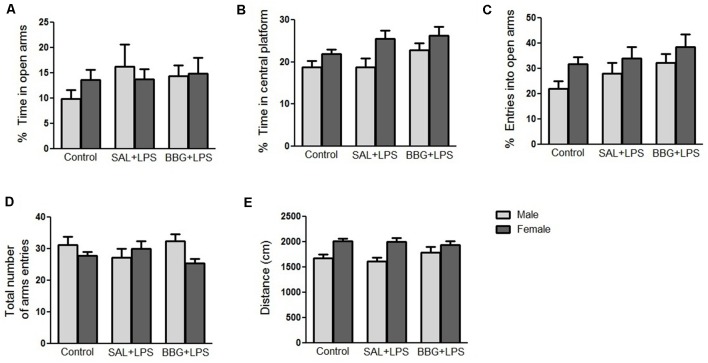
Effects of neonatal LPS and BBG-treatment on the anxiety-like behavior analyzed by elevated plus maze (EPM) test. The treatment did not show significant changes on behavioral parameters between groups and there were no interaction of group/sex: percentage of time spent in the open arms **(A)**, percentage of time spent on the central platform **(B)**, percentage of entries into open arms **(C)**, total number of arms entries (open and closed arms; **D**) and distance travaled **(E)**. Data are expressed as mean ± SEM. *p* > 0.05.

There were no significant differences in the analyzed behavioral parameters between groups [ratio of time spent in the open arms: *p* = 0.334; ratio of time spent in central platform: *p* = 0.053; ratio of entries into open arms: *p* = 0.076; total number of arms entries (open and closed arms): *p* = 0.910; traveled distance: *p* = 0.772], as well as for the interaction of groups vs. sex (*p* = 0.455; *p* = 0.522; *p* = 0.832; *p* = 0.150; *p* = 0.356; respectively; Figures 3A–E). On the other hand, we observed differences with respect to the sex of the animals to the ratio of entries into open arms (*p* = 0.023; females presented more entries when compared to males), the ratio of time spent in central platform (*p* = 0.002; females spent more time when compared to males) and the traveled distance (*p* < 0.001; females traveled a longer distance as compared to males; see Supplementary Figure S2). There were significant changes in the analyzed behavioral parameters only between the estrous cycle phases, regardless of the experimental group (see Supplementary Table S1 and Supplementary Figure S3).

#### Hot-Plate Test

**Figure 4:**
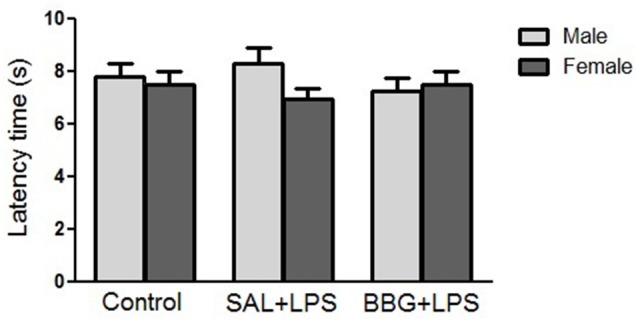
Effects of neonatal LPS and BBG-treatment on the pain sensitivity analyzed by hot-plate test. The treatment did not show significant changes on latency time between groups and there were no interaction of group/sex. Data are expressed as mean ± SEM. *p* > 0.05.

There were no significant differences in reaction latency among groups (*p* = 0.856), even between males and females (*p* = 0.310), as well as for the interaction of groups vs. sex (*p* = 0.369; Figure 4). There were no significant changes regarding the estrous cycle (see Supplementary Table S2).

#### Tail-Flick Test

**Figure 5:**
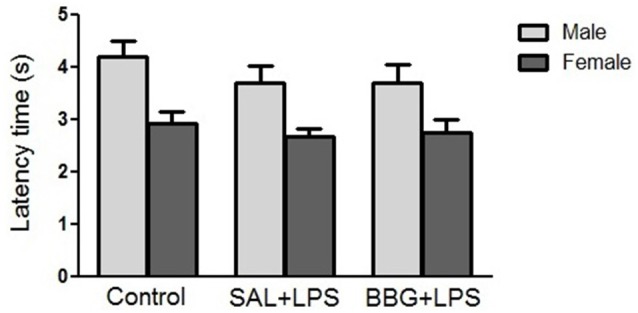
Effects of neonatal LPS and BBG-treatment on the pain sensitivity analyzed by tail-flick test. The treatment did not show significant changes in latency time between groups and there was no interaction of group/sex. Data are expressed as mean ± SEM. *p* > 0.05.

There were no significant differences in reaction latency between groups (*p* = 0.299), as well as for the interaction of groups vs. sex (*p* = 0.807; Figure 5). However, females showed a significantly lower response (*p* < 0.001) when compared to the males (see Supplementary Figure S4). There were no significant changes in reaction latency regarding estrous cycle (see Supplementary Table S3).

### Effects of Neonatal LPS and BBG-Treatment on Oxidative Stress

**Figure 6:**
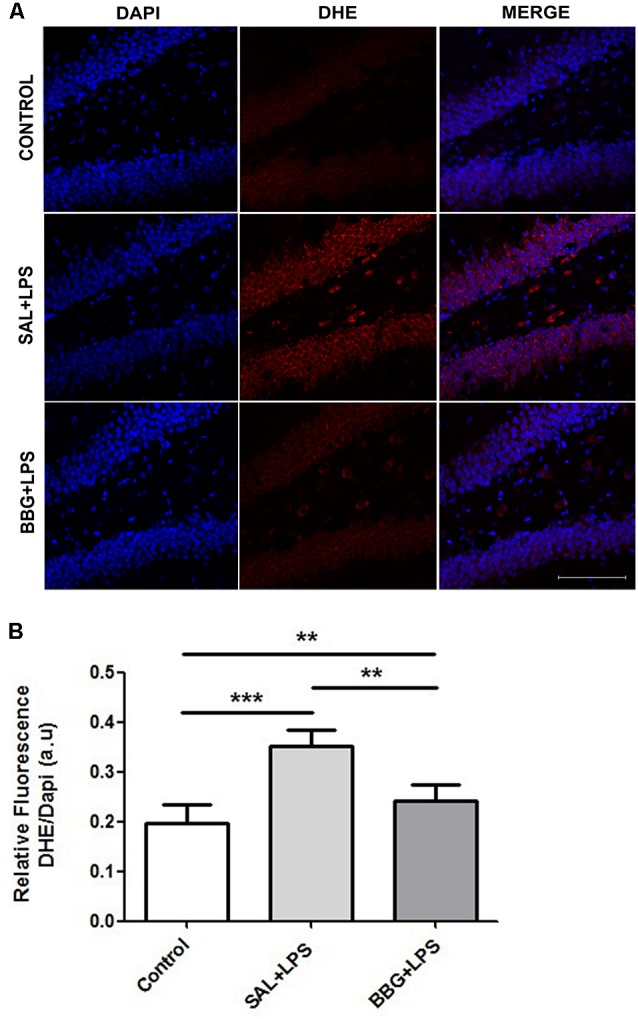
Effects of neonatal LPS and BBG-treatment on the superoxide production. Superoxide anion were detected in hippocampus of animals by confocal microscope using DHE probe and ROS level was represented by fluorescence intensity. A decrease in BBG-treated animals was observed when compared to LPS-treated animals. Confocal microscopy of hippocampus of different groups **(A)**. Quantification of superoxide anion by the analysis of pixelationv Higher level of DHE intensity was observed in the LPS-treated group and it is opposite of the result showed in the BBG group **(B)**. DHE: red spots and DAPI: all blue nucleus. Scale bar represents 100 μm. Data are expressed as mean ± S.E.M. **p < 0.01, ***p < 0.001. a.u: arbitrary units.

We observed significant differences in DHE intensity among groups (*p* < 0.001), although no difference was noted between males and females (*p* = 0.734), as well as for the interaction of groups vs. sex (*p* = 0.815). The Figure 6A shows the images of stained brain slices with DHE. The LPS-treated group exhibited higher level of DHE intensity when compared to control and BBG groups, as well as BBG group when compared to control group (Figure 6B).

**Figure 7:**
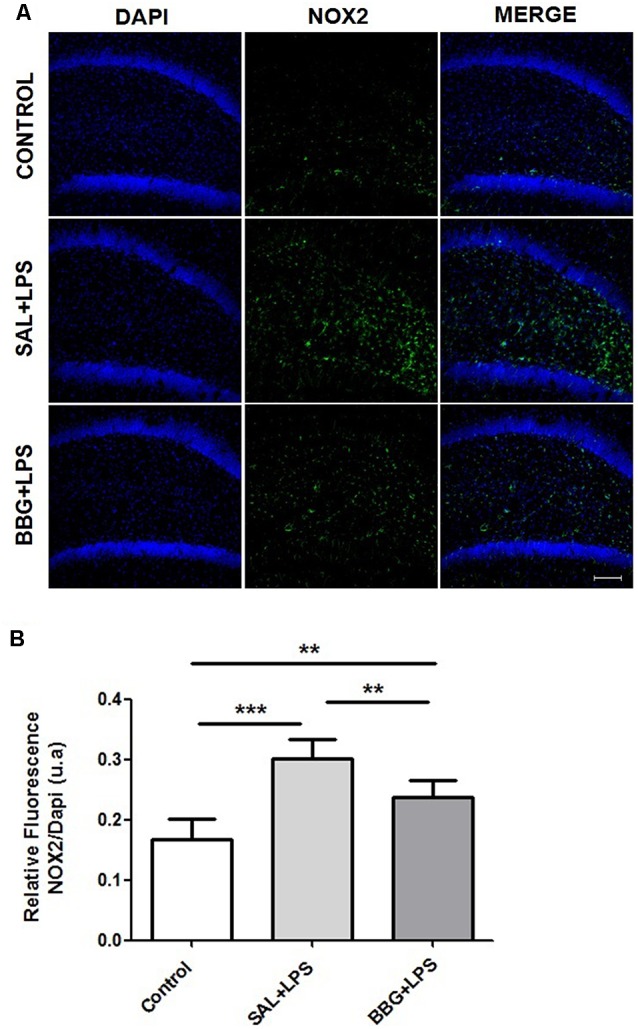
Effects of neonatal LPS and BBG-treatment on the gp91-phox/NOX2 expression. NOX2 subunit were detected in the hippocampus of animals by immunofluorescence. A decrease in BBG-treated animals was observed when compared to LPS-treated animals. Confocal microscopy of hippocampus of different groups **(A)**. Quantification of gp91-phox by the analysis of pixilation. Higher level of gp91-phox intensity was observed in the LPS-treated group. On the other hand, a decreased level was found in the BBG group. **(B)**. gp91-phox: green spots and DAPI: all blue nucleus. Scale bar represents 100 μm. Data are expressed as mean ± SEM. **p < 0.01, ***p < 0.001. a.u: arbitrary units.

Also, significant differences were found in the immunoreactivity for 91-phox/NOX2 between the groups (*p* < 0.001). The Figure 7A shows the images of stained brain with anti-gp91-phox/NOX2. The LPS-treated group exhibited higher levels of intensity for gp91-phox/NOX2 when compared to control and BBG groups, as well as BBG group when compared to control group (Figure 7B).

**Figure 8:**
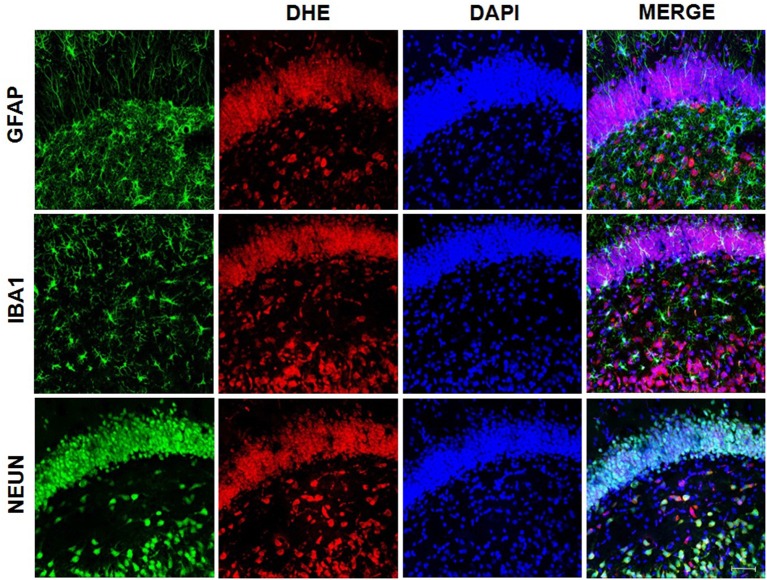
Superoxide presence on neurons. Superoxide anion was detected using DHE probe. Confocal microscopy of hippocampus showed that DHE probe overlaps with NeuN, indicating the presence of superoxide in neurons.

**Figure 9:**
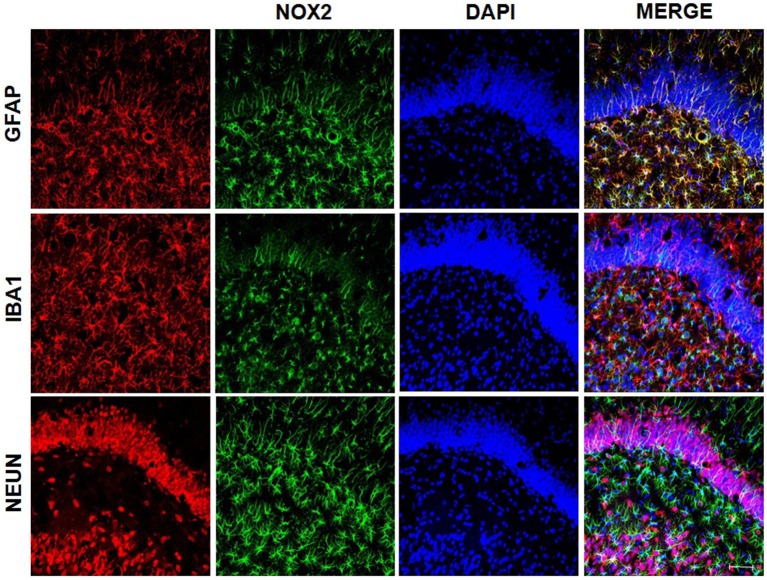
gp91-phox/NOX2 expression on astrocytes. gp91-phox was detected using immunofluorescence. Confocal microscopy of hippocampus showed colocalization with glial fibrillary acid protein (GFAP), indicating its expression on astrocytes.

We used samples from LPS group to access the presence of superoxide by double staining. DHE probe showed an overlap only with NeuN staining (Figure 8) while gp91-phox/NOX2 overlaps with the astrocyte marker, GFAP (Figure 9).

## Discussion

Neonatal inflammation affects 11% of all live births and depending on the gestational age at birth (Vergnano et al., 2011), 25%–60% of the extremely preterm infants experience at least one episode of invasive bacterial infection during their entire staying in the neonatal intensive care unit (Stoll et al., 2010). A large amount of evidence has shown that the early life exposure to inflammation has been associated with long-term neurological impairment such as motor dysfunction in childhood and psychiatric disorders in adulthood. The first week of life is characterized by great plasticity and reorganization of the CNS (Bartlett et al., 2017) and represents a critical period in which inflammatory events can have significant neurological long-term consequences. The neonatal inflammation may induce long lasting changes in the CNS functioning, as the regulation of microglial and astrocyte activity, the synthesis and secretion of cytokines; the neurotransmission and the function of the adrenal pituitary hypothalamus axis (Ren et al., 2004; LaPrairie and Murphy, 2007).

In our study, the animals exposed to the inflammatory stimulus presented a significantly higher mortality rate when compared to the control group during the first week of life. This increased mortality may be related to a strong systemic inflammatory response syndrome (SIRS) induced by the LPS, resulting in multiple organ dysfunction (Mason, 1993; Zouikr et al., 2014). SIRS is characterized by excessive systemic cytokine synthesis, damage and cell death and tissue damage (Davies and Hagen, 1997; Cauwels et al., 2014). On the other hand, animals exposed to neonatal inflammation treated with BBG had lower mortality rate. Cauwels et al. (2014) demonstrated that the removal of extracellular ATP using systemic apyrase prevented the increase of cytokines and also prevented necrosis, mitochondrial damage, apoptosis, and death of animals. Thus, systemic blockade of P2X7R with BBG may have played similar effects as the systemic apyrase in the cytokine secretion and therefore, reducing the mortality rate.

Body weight gain is also an indicator of the impact of inflammatory processes during the neonatal period. Spencer et al. (2006) demonstrated that animals exposed to an inflammatory stimulus on the PND7 had a significantly lower body weight in PND74 when compared to controls. Similar findings were observed by Hodgson et al. (2001), which demonstrated that repeated inflammatory stimuli in the neonatal period is associated to limited body weight gain in adulthood. Our results support this data, whereas LPS-treated animals showed decreased body weight compared to controls in all time points from PND10 for males and females. Here, LPS treatment clearly affected body weight gain and the association with BBG treatment was able to reverse this effect only in males from PND45, but not in females.

We believe that the decreased body weight gain in the LPS-treated rats could be related to the reduction in frequency of feeding during the first week of life when the LPS injections were administered to the pups. Also, inflammation may alter levels of leptin and neuropeptide Y, which play a critical control over satiety and body weight in the long term (Schwartz et al., 2000; Sachot et al., 2004). The neonatal period is crucial for the development of the neural circuits that control feeding, and satiety and inflammation can also change it (Elmquist and Flier, 2004; Spencer et al., 2006). The mechanisms involved in the effects of P2X7R blockade on body weight are still unclear, but sexual dimorphism, in this case, may be associated with genetic and hormonal differences (Cario-Toumaniantz et al., 1998; Heiman-Patterson et al., 2005; Novak et al., 2010).

Oxidative stress is triggered by increased production of different free radicals (Hsieh and Yang, 2013). In our study, the production of ROS was assessed by the measurement of superoxide anion in response to systemic inflammation. Considering the experimental groups, regardless of the sex of animals, we noted that the animals exposed to systemic inflammation in the neonatal period presented a significantly higher superoxide production in the dentate gyrus of hippocampus, when compared to control animals. Most importantly, our findings suggest that the blockade of P2X7R with BBG during the inflammatory process down regulated the superoxide anion production in the brain of adult animals when compared to the animals that received LPS. This finding corroborates the studies of Feng et al. (2015) and Munoz et al. (2017), which demonstrated the attenuation of free radical production by blocking the activation of P2X7R.

The relationship between oxidative stress in the CNS and systemic inflammation has been documented in the literature. Correa et al. (2013) demonstrated that 24 h after systemic administration of LPS in neonatal rats, there was an increased level of superoxide dismutase (SOD) and other proteins associated with antioxidant systems in the brain. It is known that NOX plays a significant role in the synthesis of ROS induced by the activation of P2X7R, which induces the increase of proinflammatory cytokines (Hewinson and MacKenzie, 2007; Mead et al., 2012; Jiang et al., 2015). Thus, activation of P2X7R contributes doubly to the increase of superoxide production and, consequently, may favor the maintenance of the vicious cycle of oxidative stress/inflammation/oxidative stress, which can be minimized by the use of antagonists of this receptor (Parvathenani et al., 2003; Pfeiffer et al., 2007).

In the present study, we demonstrated that the increased production of superoxide in the dentate gyrus of the hippocampus in adult rats exposed to neonatal inflammation was associated to an increased immunoreactivity for gp91-phox, the NOX2 catalytic subunit (Yu et al., 1998), which can be expressed by neuron, astrocyte and microglia (Park et al., 2007; Dohi et al., 2010; Li et al., 2014).

Each member of NOX family proteins reveals a distinct cellular and tissue distribution pattern with specific roles in the ROS production (Noguchi et al., 2008). These data corroborate with our findings, in which we observed that gp91-phox expression occurs particularly in astrocytes, although superoxide accumulation has been observed in neurons. It is known that superoxide, a diffusible messenger, can permeate cell membranes and acts as a neuron-glial transcellular signal (Atkins and Sweatt, 1999; Reyes et al., 2012; Spiers et al., 2015). On the other hand, the treatment with P2X7R antagonist leads to a decreased hippocampal production of superoxide and reduced immunoreactivity for gp91-phox. As the literature has demonstrated, this finding is consistent with the activation of the P2X7R, which is associated to an upregulation of NOX2, as suggested by Noguchi et al. (2008) and Deng et al. (2015), whereas C-terminal of P2X7R may regulate NOX2 activation.

Neonatal exposure to inflammatory process can lead to increased anxiety in adult animals, as cytokines modulate neurotransmitters turnover, hypothalamus-pituitary-adrenal axis, synaptic plasticity and neural circuits associated to emotional expression (Breivik et al., 2002; Salim et al., 2012). When comparing the different experimental groups, no significant difference was observed in the behavioral parameters evaluated in the EPM test for the LPS group as compared to the control group. This finding diverges from the study by Walker et al. (2004), who demonstrated that inflammatory stimuli on the 3rd and 5th days of life resulted in increased anxiety-associated behavior in the EPM test in PND80. However, the literature reveals controversial data. When using lower doses of LPS, where the neonatal exposure to 50 μg/Kg of LPS at PND3 and PND5, an increase in anxiety-like behavior was observed in male rats (Walker et al., 2004). In contrast, Spencer et al. (2005) reported no significant changes in anxiety levels following administration of 100 μg/Kg of LPS at PND14 in male rats. In another report, with higher LPS dose, anxiety-like behavior was decreased in female rats given 1 mg/Kg at PND5 (Wang et al., 2013). Therefore, the effect of LPS injection on anxiety behavior in EPM test is highly dependent on the dose and time of LPS administration.

When assessing the effect of the sex of the animals on the anxiety behavior, females were less anxious than males, as they spent longer in the central platform and presented a greater number of entries in the open arms, and greater locomotor activity as compared to the males. These findings are in agreement with a previous report, which suggested that female rats show less anxious behavior when compared to male rats based on their performance in an EPM test (Johnston and File, 1991). However, there was no interaction between the treatments used and the sex of the animals in this study.

The exposure to an inflammatory insult such as LPS during the first week of life is likely to interfere with the normal developmental trajectory of the nociceptive system, leading to changes in the behavioral responses following re-exposure to noxious stimuli later in life (Zouikr et al., 2014). Our results suggested that neonatal LPS injections did not affect pain sensitivity assessed by hot-plate and tail-flick tests in adulthood. However, recent studies have shown the LPS injections induce hyperalgesia (Mason, 1993; Davies and Hagen, 1997; Zouikr et al., 2014). In another study, Yirmiya et al. (1994) demonstrated that LPS-induced analgesia began about 2 h after and disappeared 30 h after the administration of LPS. Considering these controversial results, further research is required to explain these findings.

It is known that females have lower levels of stress-induced analgesia, a consequence of differences in the endogenous opioid system (Kavaliers and Innes, 1987; Wiesenfeld-Hallin, 2005). Interestingly, considering only the sex independently of the experimental groups, there was no significant difference in latency between males and females in the hot-plate test. On the other hand, in the tail-flick test, females showed a significantly lower latency compared to males. However, we also did not observe interaction between the treatments used and the sex of the animals in both tests.

Although the primary function of the hippocampus is learning and memory (Eichenbaum et al., 1992), it is also associated with emotions and nociception (Ploghaus et al., 2001; McHugh et al., 2004; Liu and Chen, 2009; Vachon-Presseau et al., 2013). In this sense, evidence begins to appear that increased oxidative stress in the hippocampus may at least have relevance in triggering anxious behavior (de Oliveira et al., 2007). Considering the fact that the animals of the LPS group showed higher levels of oxidative stress than the animals of other experimental groups, it is interesting that they did not present alterations in behaviors associated with anxiety or in the sensitivity to pain once several studies suggest that oxidative stress has great relevance in triggering anxiety disorders and in altering nociception (de Oliveira et al., 2007; Bouayed et al., 2009; Raut and Ratka, 2009).

As already mentioned, our data diverges from the literature. We developed a chronic protocol, using several doses of LPS during an extended treatment time. On the other hand, previous reports usually refer to acute treatments, done with one or two doses of LPS injection, which interfered significantly in the results of anxiety and nociception presented, but even in literature, the results are paradoxical. Therefore, it is important to highlight that age and the LPS dose are critical in generating conflicting outcomes. These models have been designed for a clear understanding of the human condition, which leads to extensive variations in many studied parameters.

Our findings suggest critical functions of P2X7R-mediated oxidative stress and mortality. Importantly, inhibition of P2X7R by BBG has been found effective in reducing superoxide production that leads to inflammatory responses and mortality rate, conferring neuroprotection in adulthood. Furthermore, our study proposes that pharmacological modulation of P2X7R mediated by its antagonist may represent a potential therapeutic target in neurological diseases.

## Ethics Statement

This study was carried out and approved in accordance with the recommendations of “Ethics Committee of UNIFESP.”

## Author Contributions

CS conceived the study, carried out the laboratory experiments, behavioral tests, analyzed the data and performed the statistical analysis. JP and SR assisted drugs injection, behavioral tests, data collection and formatted the references. AM carried out the brain samples and helped to draft the article. MC performed immunofluorescence experiments, photographed, analyzed the images and helped to draft and critically revised the article. LM contributed with the study design and the article revision and interpreted the results. AL contributed with the study design, the data analysis, reagents, materials and critically revised the article. The work presented here was carried out in collaboration between all authors. All authors reviewed and approved the final manuscript.

## Conflict of Interest

The authors declare that the research was conducted in the absence of any commercial or financial relationships that could be construed as a potential conflict of interest.
