# Supplementary material for: LPS-Induced Systemic Neonatal Inflammation: Blockage of P2X7R by BBG Decreases Mortality on Rat Pups and Oxidative Stress in Hippocampus of Adult Rats
Source: Front Behav Neurosci. 2019 Nov 6;13:240. doi: 10.3389/fnbeh.2019.00240 (PMC6878118; doi:10.3389/fnbeh.2019.00240)
Supplement: Supplementary file 1 [file Data_Sheet_1.docx]

# SUPPLEMENTAL DATA

**1. Material and Methods**

**1.1. Estrous cycle monitoring**

In order to verify the influence of estrous cycle on treatment, female rats were evaluated according to the stage of the estrous cycle. The animals were submitted daily to vaginal smears from PND66 until PND84 for determination of different phases of estrous cycle. Animals were assigned to three groups: proestrus, diestrus and estrus. The biological sample was analyzed and photographed under an optical microscope (Nikon, model Eclipse 50i) with a digital camera (Nikon, model DXM1200) and ACT-1 (Nikon, version 2.7).

**2. Results**

# 2.1. Sickness of animal monitoring

The LPS-treated pups demonstrated a “sick” appearance when compared to control and BBG+LPS groups (Figure S1).


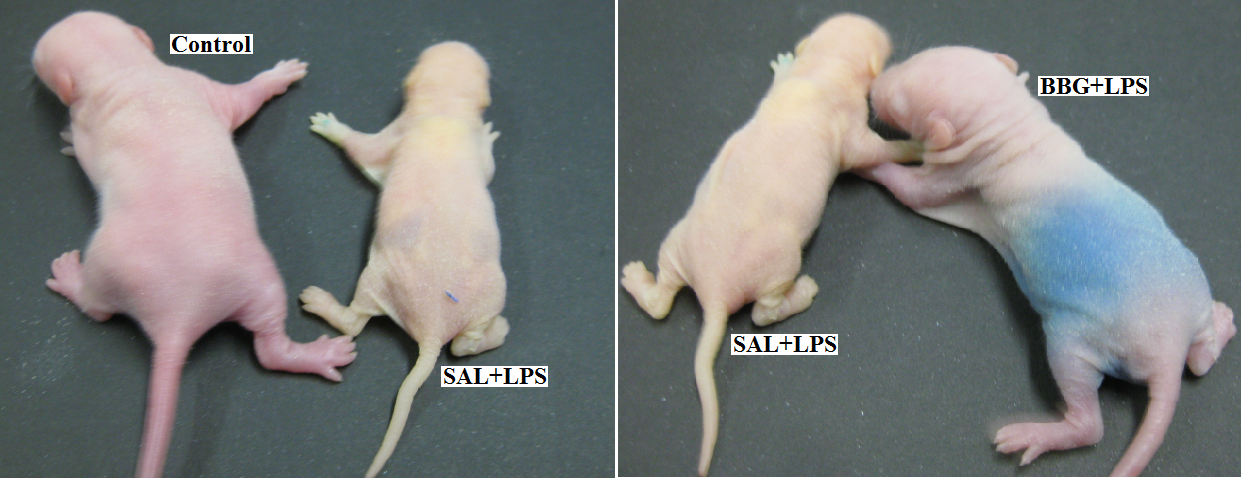


**Figure S1: Physical aspect of control and treated pupies.** The LPS-treated animal presents pallor and poor growth when compared to control and BBG-treated groups. SAL: saline; LPS: lipopolysaccharide; BBG: brilliant blue G.

**2.2. Effects of neonatal LPS and BBG-treatment on the behavioral and nociceptive**

**2.2.1. Influence of the sex on the elevated plus-maze test**

Comparison between sexes shows that female rats presented higher percentage on the entries into the open arms, time spent on the platform and distance travelled (Figure S2).

**
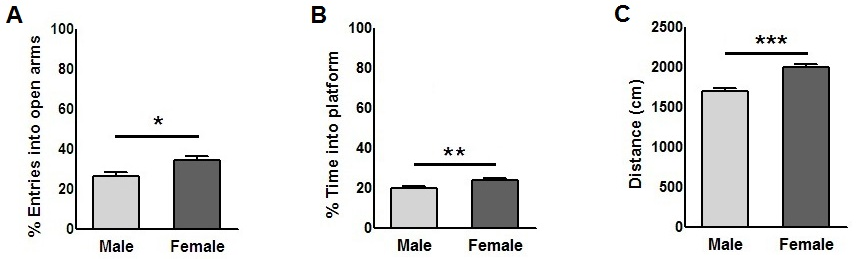
**

**Figure S2: Influence of the sex on the anxiety-like behavior analyzed by elevated plus‑maze test**. Entries into the open arms **(A)**, time spent on the platform **(B)** and distance travelled **(C)**. The comparison between the sex showed that males travelled shorter distance than females. Data are expressed as mean ± S.E.M. **p < 0.05, **p < 0.01, ***p < 0.001.*

**2.2.2. Influence of the estrous cycle on the elevated plus-maze test**

There were no significant changes in analyzed behavioral parameters between groups (females only; ratio of time spent in the open arms: p = 0.890; ratio of time spent in central platform: p = 0.061; ratio of entries into open arms: p = 0.299; total number of arms entries [open and closed arms]: p = 0.187; travelled distance: p = 0.993), as well as for the interaction of groups versus estrous cycle (p = 0.792; p = 0.582; p = 0.683; p = 0.739; p = 0.389; respectively) (Table S01). However, we observed differences with respect to the estrous cycle phases to the ratio of entries into open arms (p = 0.038; females in proestrous [42.8±3.5%] presented more entries when compared to females in estrous [25.2±4.7%]) (Figure S3).

| **Elevated Pluz Maze Test (mean±S.E.M)** | | | | | | | |
| --- | --- | --- | --- | --- | --- | --- | --- |
| **Gruoup** | **Cycle phase** | **n** | **%TOA** | **%TSCP** | **%EOA** | **TAE** | **D (cm)** |
| Control | Proestrous | 6 | 16.52±5.09 | 23.86±1.58 | 39.76±6.23 | 26.67±1.26 | 1883±55 |
|  | Estrous | 4 | 11.47±4.44 | 21.33±3.27 | 20.46±4.29 | 33.50±4.17 | 1998±159 |
|  | Diestrous | 10 | 12.76±2.31 | 21.03±1.55 | 31.45±3.04 | 26.20±1.32 | 2107±70 |
| SAL+LPS | Proestrous | 3 | 18.93±1.99 | 25.32±4.63 | 48.69±6.16 | 32.00±1.00 | 1944±63 |
|  | Estrous | 3 | 6.06±3.35 | 25.95±4.98 | 21.82±7.70 | 33.33±9.67 | 1986±288 |
|  | Diestrous | 6 | 15.16±2.38 | 25.29±2.75 | 32.70±5.81 | 27.33±1.82 | 2027±105 |
| BBG+LPS | Proestrous | 3 | 15.87±3.40 | 29.89±0.60 | 42.81±2.52 | 22.67±0.88 | 1862±66 |
|  | Estrous | 2 | 15.41±1.32 | 31.35±5.40 | 39.66±15.52 | 29.00±0.00 | 2244±136 |
|  | Diestrous | 6 | 14.24±5.68 | 22.68±2.94 | 35.99±8.47 | 25.67±2.29 | 1884±95 |

**Table S1: Data of anxiety-like behavior of elevated plus-maze test considering group/estrous cycle phase.** The treatment with LPS and/or BBG did not show significant changes on behavioral parameters between groups and there were no interaction of group/estrous cycle phase. Each value represents the means ± S.E.M (standard error of the mean) of the values obtained from female animals. LPS: lipopolysaccharide; BBG: brilliant blue G; n: number of animals; % TOA: percentage of time spent in the open arms; % TSCP: percentage of time spent in central platform; % EOA: percentage of entries into open arms; TAE: total number of arms entries (open and closed arms); D: distance travalled (centimeters)*.*

**Figure S3: Influence of the estrous cycle on the percentage of entries into the open arms of an elevated plus-maze.** The analysis of the percentage of entries into the open arms between estrous cycle phases show a decreased frequency in animals in estrous phase compared to proestrus. Data are expressed as mean ± S.E.M (standard error of the mean) of the values obtained from female animals. **p < 0.05.*

**2.2.3. Influence of the estrous cycle on the hot-plate test**

There were no significant changes in reaction latency (groups [females only]: p = 0.607; estrous cycle: p = 0.654; groups versus estrous cycle: p = 0.435) (Table S02).

| **Hot-Plate Test (mean±S.E.M)** | | | |
| --- | --- | --- | --- |
| **Group** | **Cycle phase** | **N** | **Latency(s)** |
| Control | Proestrous | 7 | 8.51±0.62 |
|  | Estrous | 4 | 7.10±0.70 |
|  | Diestrous | 9 | 6.94±0.86 |
| SAL+LPS | Proestrous | 4 | 6.45±0.60 |
|  | Estrous | 4 | 6.78±0.55 |
|  | Diestrous | 4 | 7.58±1.02 |
| BBG+LPS | Proestrous | 5 | 7.38±0.90 |
|  | Estrous | 4 | 7.08±0.84 |
|  | Diestrous | 2 | 8,60±0.70 |

**Table S2: Data of latency of hot-plate test considering group/estrous cycle phase.** The treatment with LPS and/or BBG did not show significant changes on pain measured by latency time between groups and there were no interaction of group/estrous cycle phase. Each value represents the mean ± S.E.M (standard error of the mean) of the values obtained from female animals. LPS: lipopolysaccharide; BBG: brilliant blue G; s: seconds; n: number of animals.

**2.2.4. Influence of the sex on the tail-flick test**

Comparison between sexes shows that female rats had a lower time of latency to remove the tail after painful stimulus (Figure S4).

*
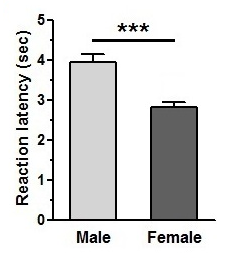
*

**Figure S4: Influence of the sex on the pain sensitivity analyzed by tail-flick test**. Females had a lower time of latency to remove the tail after painful stimulus. Data are expressed as mean ± S.E.M. ****p < 0.001.*

**2.2.5. Influence of the estrous cycle on the tail-flick test**

There were no significant changes in reaction latency (groups [females only]: p = 0.574; estrous cycle: p = 0.285; groups versus estrous cycle: p = 0.853) (Table S3).

| **Tail-Flick Test (mean±S.E.M)** | | | |
| --- | --- | --- | --- |
| **Group** | **Cucle phase** | **N** | **Latency (s)** |
| Control | Proestrous | 6 | 2.71±0.29 |
|  | Estrous | 6 | 2.73±0.30 |
|  | Diestrous | 8 | 3.23±0.45 |
| SAL+LPS | Proestrous | 7 | 2.49±0.19 |
|  | Estrous | 2 | 3.10±0.19 |
|  | Diestrous | 3 | 2.82±0.40 |
| BBG+LPS | Proestrous | 2 | 2.31±0.65 |
|  | Estrous | 2 | 2.17±0.10 |
|  | Diestrous | 7 | 3.05±0.33 |

**Table S3: Data of latency of tail-flick test considering group/estrous cycle phase.** The treatment with LPS and/or BBG did not show significant changes on pain measured by latency time between groups and there were no interaction of group/estrous cycle phase. Each value represents the mean ± S.E.M (standard error of the mean) of the values obtained from female animals. LPS: lipopolysaccharide; BBG: brilliant blue G; s: seconds; n: number of animals.
